# A systematic bibliometric analysis on the clinical practice of CGM in diabetes mellitus from 2012 to 2022

**DOI:** 10.3389/fendo.2023.1229494

**Published:** 2023-09-22

**Authors:** Laixi Kong, Bei Deng, Maoting Guo, Mengjie Chen, Xiaoxia Wang, Mingjiao Zhang, Hongxia Tang, Qin Wang, Liwei Yang, Zhenzhen Xiong

**Affiliations:** School of Nursing, Chengdu Medical College, Chengdu, Sichuan, China

**Keywords:** diabetes mellitus, continuous glucose monitoring, bibliometric analysis, Citespace, VOSviewer

## Abstract

**Background:**

Continuous glucose monitoring (CGM) has revolutionized diabetes management, but a comprehensive analysis of its clinical implementation is lacking. This study aims to explore CGM in diabetes practice over the past decade using bibliometric analysis. It will identify trends, research focal points, and provide a framework for future investigations.

**Materials and methods:**

The Web of Science Core Collection (WOSCC) was utilized to acquire literature pertaining to the employment of continuous glucose monitoring (CGM) in diabetes that was published between the years 2012 and 2022, and to conduct a comprehensive analysis of the associated citation data. To achieve bibliometric visualization and analysis of the collated data, the bibliography package in the Rstudio(v.4.2.2), Citespace 6.2.R4, and VOS viewer were employed.

**Results:**

A total of 3024 eligible publications were extracted from 91 countries, with the United States being the leading country in terms of the number of issued articles. Furthermore, the annual publication rate has shown a gradual increase during the past decade. Among the various journals in this field, DIABETES TECHNOLOGY & THERAPEUTICS was identified as the most highly cited one. Keyword clustering analysis of the extracted publications indicates that the research hotspots in the past decade have primarily focused on “continuous glucose monitoring”, “glycemic variability”, “type 1 diabetes”, “hypoglycemia”, and “glycemic control”. Moreover, the analysis of keyword emergence reveals that “Time In Range” and “Young Adult” represent the current research frontiers for the years 2012-2022.

**Conclusion:**

The concept of Time in Range (TIR) has garnered considerable attention as a significant area of inquiry and an emerging research trend in the clinical practice of Continuous Glucose Monitoring (CGM) for Diabetes Mellitus. Moreover, recent investigations have demonstrated a growing focus on young adults with type 1 diabetes as the research population of interest. In the foreseeable future, research endeavors will persist in the pursuit of improving glycemic management among young adults through the utilization of continuous glucose monitoring (CGM) technology, while also delving into the examination of the Time in Range metric via supplementary clinical investigations.

## 1 Introduction

Diabetes, a chronic non-communicable disease, ranks third after cardiovascular and cerebrovascular diseases and tumors in posing a serious risk to human health. With the accelerating pace of urbanization, lifestyle modifications, and the aging of the population, the prevalence of diabetes is escalating rapidly. According to the International Diabetes Federation (IDF), the global prevalence of diabetes among individuals aged 20 to 79 years is estimated to be 10.5% (536.6 million people) in 2021, and this figure is projected to reach 12.2% (783.2 million people) by 2045. The global health expenditure associated with diabetes is also projected to rise continuously (1, 2). Blood glucose serves as the fundamental source of energy in the body and plays a pivotal role in maintaining normal physiological functions. Abnormal fluctuations in blood glucose levels are closely linked to the onset and progression of numerous diseases, such as diabetes mellitus and hypoglycemia. Consequently, blood glucose monitoring has emerged as a crucial and indispensable tool in clinical management. Moreover, with the growing focus on health, an increasing number of individuals are becoming aware of their blood glucose levels and adopting corresponding measures to safeguard their well-being. Continuous glucose monitoring (CGM) is a non-invasive technique that enables the continuous monitoring of the concentration of glucose in subcutaneous interstitial fluid through the use of a glucose sensor. This technology facilitates the recording of the trend and characteristics of blood glucose fluctuations in real time (3, 4). Scanning CGMs, in particular, can provide continuous glucose monitoring for up to 14 days, with the sensor measuring glucose levels every minute and storing readings every 15 minutes. Scanning allows for the presentation of continuous and reliable information on blood glucose fluctuations throughout the day. The CGM system is factory-calibrated, eliminating the need for frequent finger-stick blood calibrations during use. This feature reduces the discomfort of blood collection, promotes patient compliance and initiative in blood glucose monitoring (5), and facilitates ease of operation. With the proliferation of continuous glucose monitoring (CGM) in clinical practice, it has emerged as a widely utilized tool for ambulatory glucose monitoring, facilitating the monitoring of blood glucose levels and the identification of uncontrolled hyperglycemia, hypoglycemia, and fluctuations in blood glucose (6). Consequently, prospective clinical studies have increasingly adopted CGM devices to gather data and evaluate the blood glucose profiles of study participants, in conjunction with HbA1c findings, in order to further assess the efficacy of therapeutic interventions on HbA1c (7). Given the expanding evidence supporting the efficacy of CGM in diabetes treatment and its rising demand in primary care, it is imperative to attend to its clinical use for diabetes (8). In light of these contextual factors, this research delves comprehensively into the clinical practice of Continuous Glucose Monitoring (CGM) within the domain of diabetes. This includes investigating its impact on glycemic control, the utilization of CGM-related metrics, remote monitoring and telemedicine applications, artificial pancreas(closed-loop systems), as well as integration with insulin pump mechanisms, among other facets (9).The vast quantity of research-related literature currently being produced presents a challenge for traditional literature analysis in obtaining comprehensive and pertinent information. Bibliometric analysis, however, enables both quantitative and qualitative information contained within journal articles to be analyzed (10). This approach has been proven effective in identifying emerging topics and research frontiers across a wide range of disciplines (11, 12). Accordingly, in this study, we employ scientific bibliometric analysis to systematically examine published works, with the aim of revealing annual publication outputs, identifying leading countries, regions, journals, and institutions, and evaluating research impact. We further report on the research impact of countries, regions, institutions, and journals through analysis of keywords and co-cited literature. Finally, we explore current research hotspots and future trends in the use of CGM in clinical practice for diabetes.

## 2 Materials and methods

### 2.1 Extraction of citation data

On the 1st of August 2023, a comprehensive search was conducted on the Web of Science Core Collection (WOSCC) to retrieve all citations published from the 1st of January 2012 to the 31st of December 2022. The search was executed using the following formula: TS=(“Continuous blood glucose monitoring” OR “Continuous glucose monitoring” OR “Implantable CGM system” OR CGM OR FGM OR “Flash glucose monitoring” OR “Ambulatory glucose monitoring” OR “Continuous glucose sensors” OR “Real-time glucose monitoring” OR rtCGM OR “Subcutaneous glucose monitoring” OR “Continuous glucose measurement” OR “Continuous glycemic monitoring” OR “Continuous glucose sensing” OR “Continuous glucose meters”) AND TS=(Diabetes* OR “Diabetes mellitus*”), while limiting document types to “Article” or “Review Article”. Articles and reviews written in English were considered, while meeting abstracts, early access articles, editorial material, letters, collections, proceeding papers, news items, book chapters, hardware reviews, and withdrawn publications were excluded. The initial screening process yielded a total of 3680 original English articles, comprising 3207 articles and 473 reviews, which were deemed to be potential candidates for inclusion in the study.

**Figure 1:**
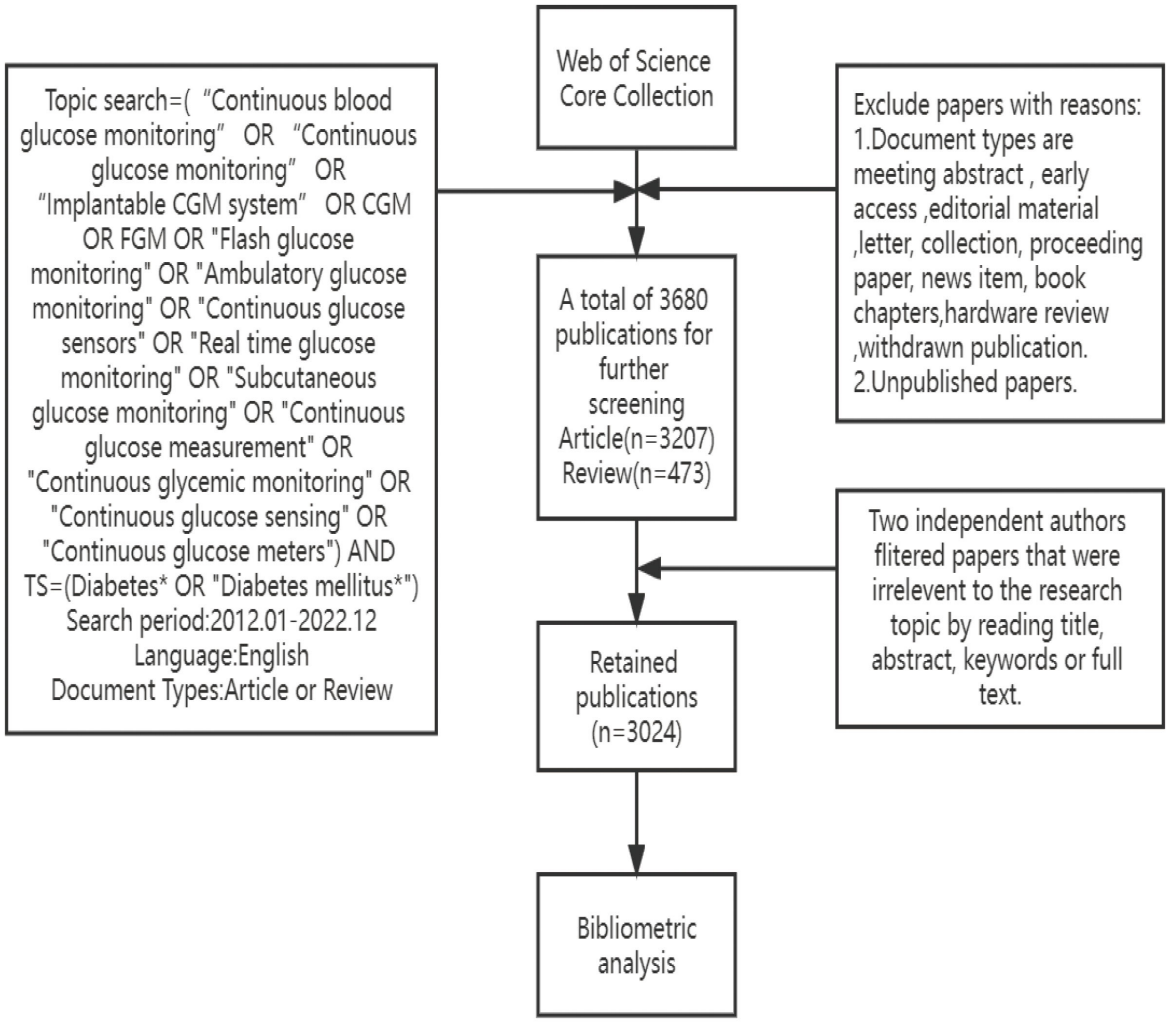
The framework diagram illustrates the comprehensive screening methodology employed in the evaluation of research literature pertinent to the clinical practice of Continuous Glucose Monitoring (CGM) in Diabetes Mellitus from 2012-2022.

To ensure the precision and caliber of the acquired data, a dual review process was undertaken by two researchers, Laixi Kong and Maoting Guo, who independently scrutinized the abstracts and keywords of literature to obtain the most pertinent articles. The objective of this study was to investigate the clinical practice of CGM in diabetes; thus, these clinical practice encompass various aspects: improvements in blood glucose control following CGM use, the utilization and interpretation of CGM-related metrics, remote monitoring and telemedicine, artificial pancreas (closed-loop systems), and the integration of CGM with multiple insulin pump systems. Exclusion criteria encompass topics such as technical design in CGM sensors, sensor material research, and unrelated reviews. Following manual screening, a total of 3024 papers were deemed suitable for inclusion in this study. From each publication, the title, publication year, country or region, institution, author, journal, references, author and keywords were methodically extracted. Further details pertaining to the literature extraction process are presented in **Figure 1**.

### 2.2 Statistical analysis

Initially, a basic statistical analysis of the dataset was conducted utilizing Rstudio (v.4.2.2). The “bibliometrix” format was employed to store the data, and the “biblishiny” package was utilized to extract a range of features related to the research literature between 2012 to 2022 (13). These features, including Main Information, Most Relevant Authors, Author’s Production Over Time, Most Global Cited Documents, served for quantitative analysis. Subsequently, CiteSpace 6.1.R6 was utilized to cluster the keywords of institutions present in the literature, perform dual map overlay analysis of journals, unveil keyword clustering analyses within the text, identify the strongest cited bursts, and construct co-cited references timeline maps of publications. In addition, VOS viewer was employed to identify the collaborative networks of countries and institutions, evaluate the keywords pertaining to the subject, and visualize the post-analysis of the results.

## 3 Results

### 3.1 Publications

**Figure 2:**
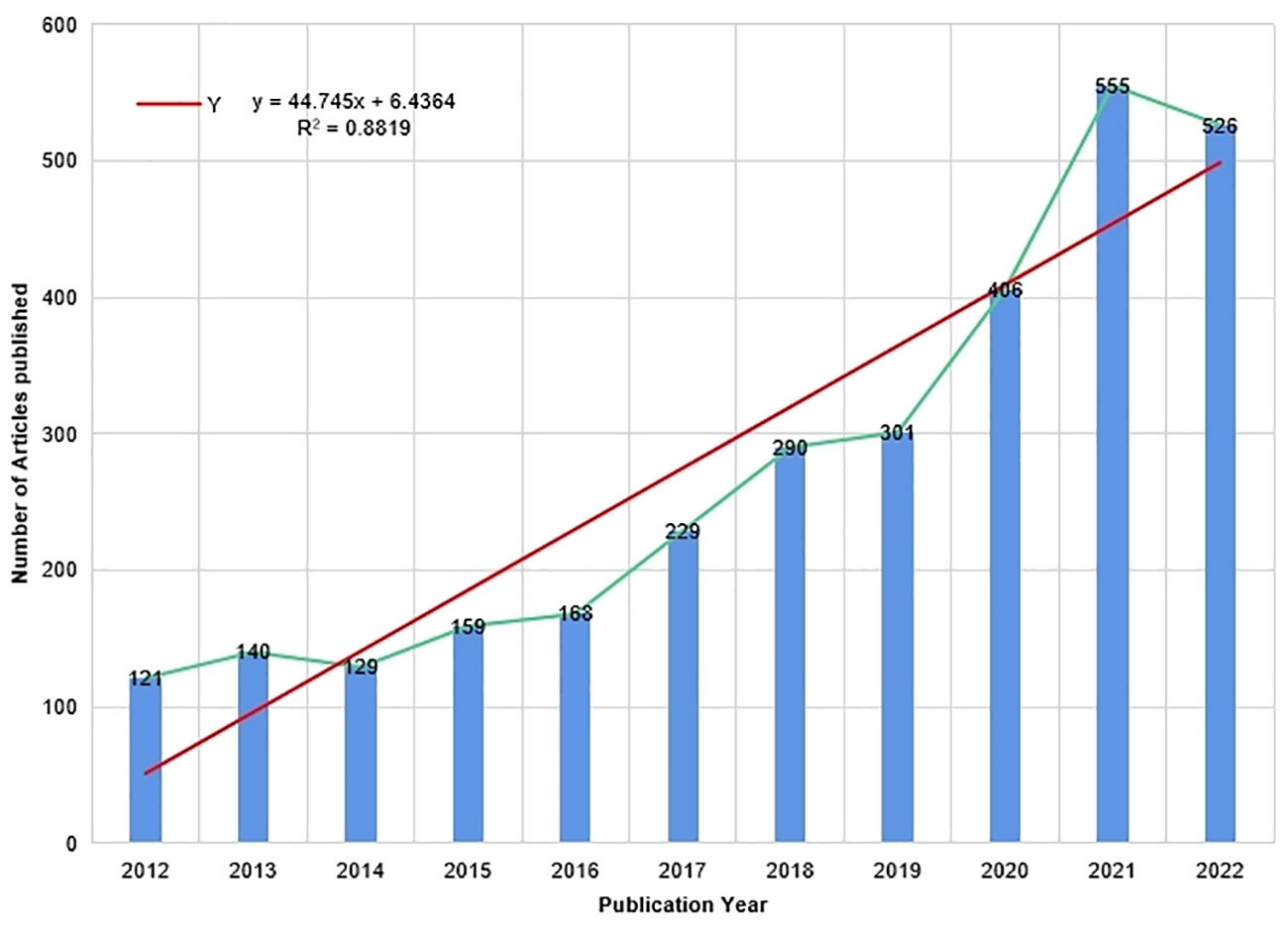
Trends in the Number of Publications on the Clinical Practice of CGM in Diabetes Mellitus from 2012 to 2022.

**Figure 3:**
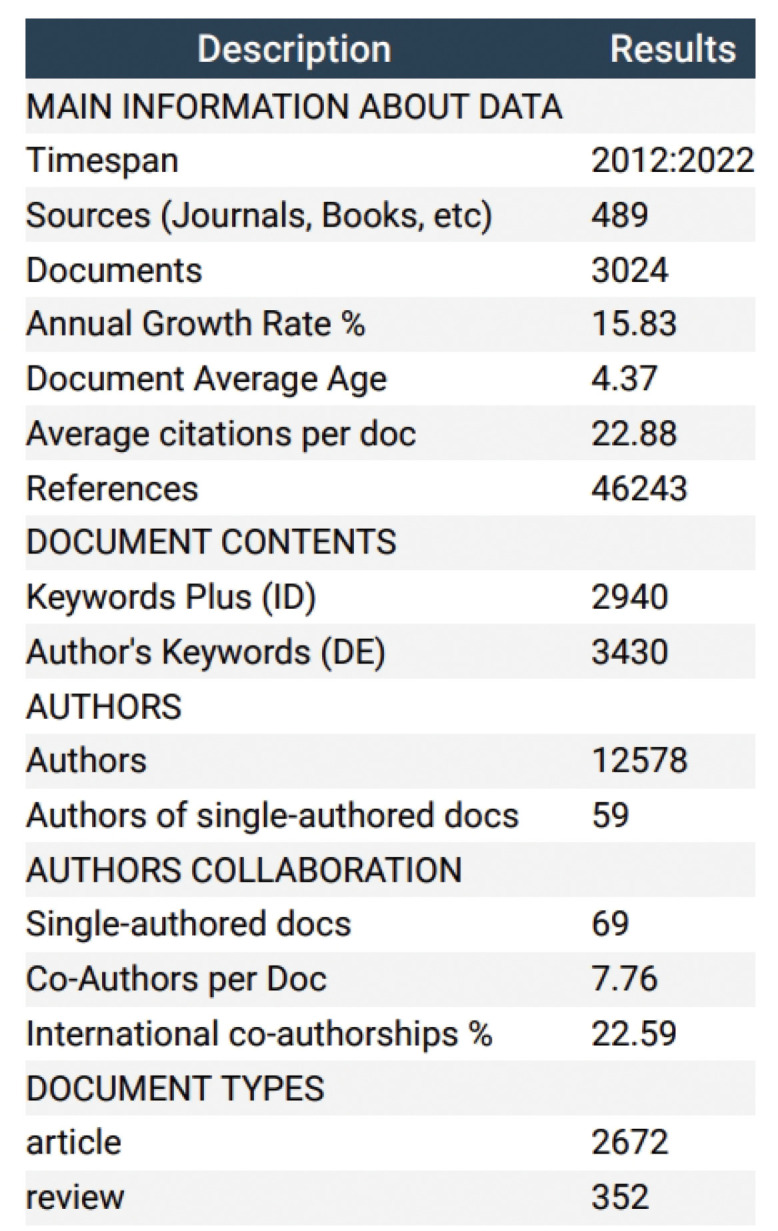
Main information about all Related Articles from 2012 to 2022.

In this review, a comprehensive analysis of 3024 literature sources was conducted, and the resulting search data was used to plot the trends in studies related to the application of continuous glucose monitoring (CGM) to clinical practice in diabetes using R studio. As illustrated in **Figure 2**, the analysis revealed a consistent annual increase in the volume of research articles on this subject from 2012 to 2019, followed by a sharp rise in the number of publications from 2019 to 2021, suggesting a heightened interest in research pertaining to clinical practice of CGM in diabetes during this period. Notably, 2021 recorded the highest output of 555 articles. Furthermore, a linear trend line of annual publications was developed to gain further insights into the output trend, resulting in the equation Y=44.745X+6.4364, where Y represents the annual publications and X denotes the year. This model exhibits a coefficient of determination (R²) of 0.8819. **Figure 3** presents an overview of the analyzed articles, encompassing a total of 46243 references and an average publication year of 4.37. Moreover, each article garnered an average of 22.88 citations, while the annual publication growth rate was 15.83%.

### 3.2 Countries and regions

**Figure 4:**
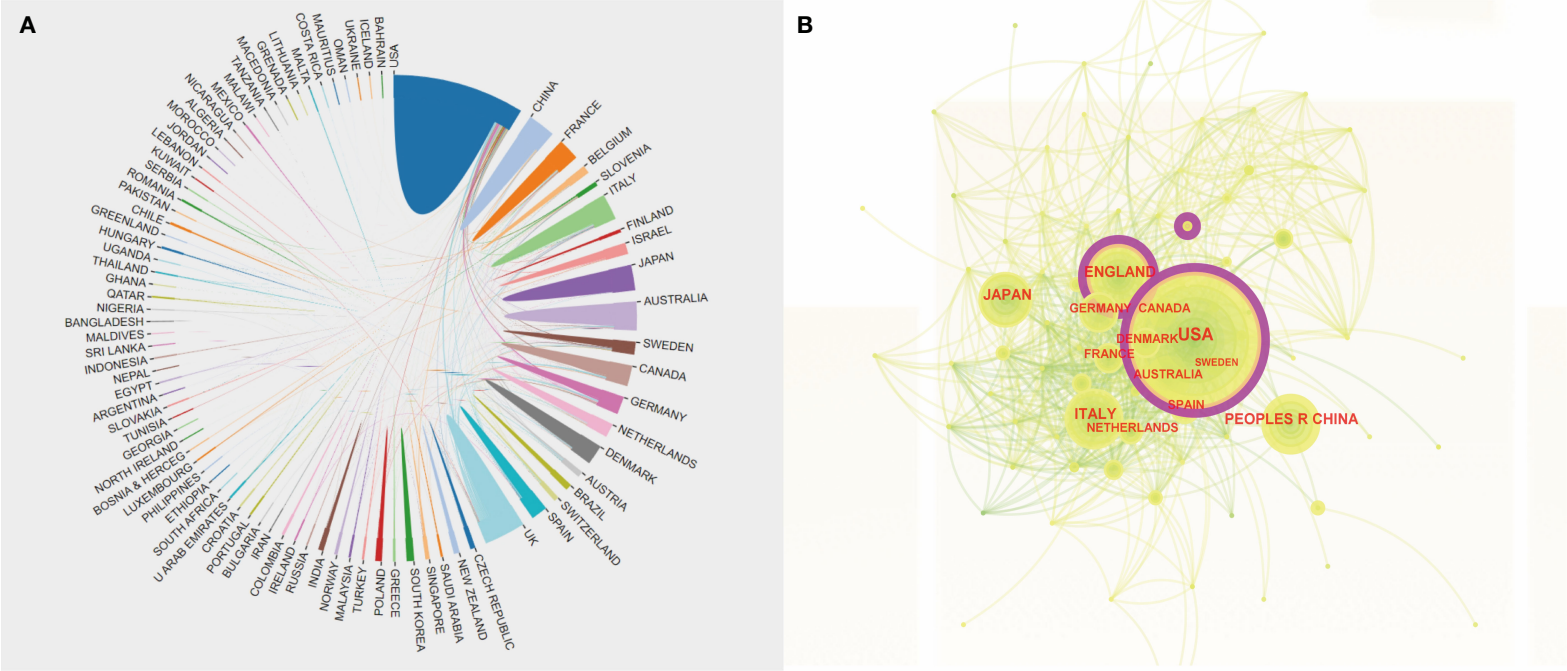
**(A)** An Analysis of International Cooperation Between Diverse Countries. The correlation among distinct color blocks signifies the bilateral collaborative association between the two countries. **(B)** Cooperation of Countries or Regions that Contributed to Publications on the Clinical Practice of CGM in Diabetes Mellitus from 2012 to 2022. The size of the purple ring area serves as an indicator of the scope of influence of the regional articles and is equivalent to their centrality.

A total of 91 countries have conducted studies on the topic at hand. The **Figure 4A** indicated that the United States had the highest number of articles published (1029), followed by the United Kingdom (331) and China (264). The top 10 countries in terms of output were summarized in the **Table 1**, with the United States exhibiting the highest centrality (0.17), H-index (86), and Citations Per Papers (32.00), surpassing other countries by a significant margin. Although China and Japan ranked high, their H-index and centrality were comparatively lower than those of other countries. The international cooperation relationship of each country was visualized using the CiteSpace, as shown in **Figure 4B**, where nodes represented countries and node size reflected the amount of national issuance. The purple portion of the circle represented centrality, with the United States positioned at the center, indicating frequent cooperation with other countries. Furthermore, the circle of the United States was the largest, indicative of the most influential issuance in the region.

**Table 1:** Top 10 countries or regions with publications on clinical practice of CGM in diabetes mellitus from 2012 to 2022.

Rank	Country/Region	Count	Centrality	H-index	Citations Per Papers
1	USA	1029	0.17	86	32.00
2	England	331	0.15	47	26.42
3	China	264	0.00	28	12.07
4	Japan	261	0.00	27	11.86
5	Italy	253	0.10	42	21.30
6	Germany	183	0.08	40	34.72
7	Australia	173	0.09	35	17.08
8	France	142	0.05	36	18.36
9	Canada	141	0.03	31	27.68
10	Denmark	127	0.07	31	20.26

### 3.3 Institutions

**Table 2** illustrates the top 10 institutions with the highest literature output, where in HARVARD UNIVERSITY, UNIVERSITY OF COLORADO SYSTEM, and UNIVERSITY OF COLORADO ANSCHUTZ MEDICALCAMPUS emerged as the top three institutions with the highest number of published articles (151, 144, and 132, respectively). Notably, HARVARD UNIVERSITY exhibited a significantly higher H-Index compared to other two institutions, indicating its dominant influence in publishing articles. Seven out of the top 10 institutions were affiliated with the United States.

**Table 2:** The top 10 institutions with publications on clinical practice of CGM in diabetes mellitus from 2012-2022.

Rank	Institutions	Counts	H-Index	Countries or Regions
1	HARVARD UNIVERSITY	151	41	America
2	UNIVERSITY OF COLORADO SYSTEM	144	38	America
3	UNIVERSITY OF COLORADO ANSCHUTZ MEDICALCAMPUS	132	36	America
4	STANFORD UNIVERSITY	111	39	America
5	JAEB CENTER FOR HEALTH RESEARCH	103	45	America
6	N8 RESEARCHPARTNERSHIP	97	33	England
7	UNIVERSITY OF CAMBRIDGE	94	31	England
8	UNIVERSITY OF COPENHAGEN	84	29	Denmark
9	HARVARD MEDICAL SCHOOL	77	31	America
10	JOSLIN DIABETES CENTER INC	77	32	America

**Figure 5:**
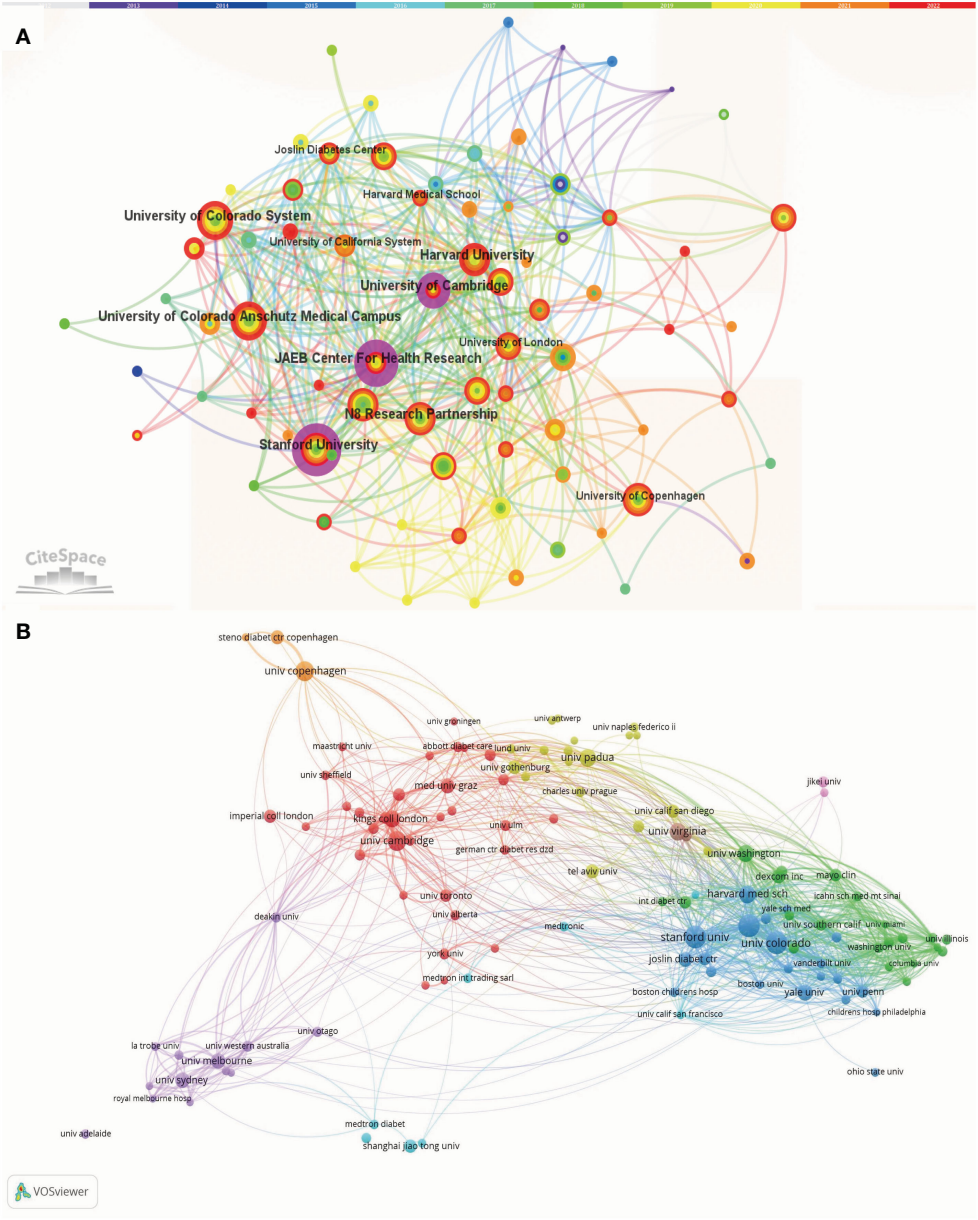
**(A)** Collaborative Network Analysis by CiteSpace Amongst Institutions Pertaining to the Clinical Practice of CGM in Diabetes Mellitus from 2012 to 2022. Each node with colorful annual rings represents an institution, and the size of each node represents its relative quantity of research output. **(B)** The overlay visualization map of Institution co-authorship analysis conducted by VOSviewer.

The collaborative relationships between institutions were disclosed through the use of CiteSpace, as depicted in **Figure 5A**. The connecting line between each of the two labels in **Figure 5B** shows that the institutions in the same country cooperate closely.

### 3.4 Analysis of authors

**Figure 6:**
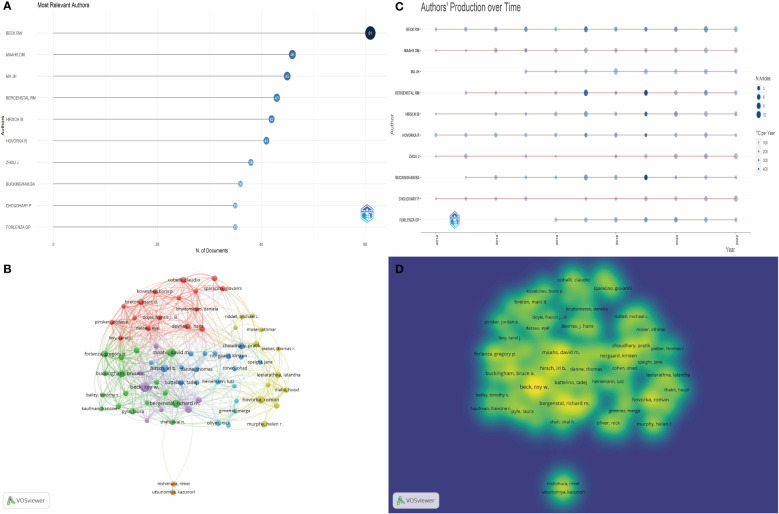
**(A)** Top 10 most relevant authors in this field. **(B)** The visual depiction of author co-citation analysis using VOSviewer showcases a graphical representation where each author is symbolized as a distinct node. The size of these nodes is proportionate to the total number of citations received. Connections between nodes signify instances of co-citation, implying a connection between the authors' works. The proximity between nodes reflects their degree of association, with shorter distances denoting a stronger relationship. Nodes with closer proximity are grouped together using matching colors, indicating their membership in a shared cluster. **(C)** Top 10 authors’ production over time. The circle size represents the number of documents (N. Documents), and the shade of the color signifies the total number of citations (TC). **(D)** The visualization of author collaboration patterns produced by VOSviewer depicts clusters denoting groups of authors with significant collaborative ties. Authors closely linked in terms of cooperation are represented within a shared cluster, visually distinguished by a common color.

**Figure 6A** presents the roster of the ten most pertinent authors within this research domain, with particular emphasis on the 61 articles affiliated with Roy W. Beck. A nuanced comprehension of the potency of influence and the yearly evolution of publications among these ten authors over the past decade is facilitated by **Figure 6B**. Evidently, Roy W. Beck sustains a conspicuously high echelon of scientific impact within this research sphere (14). Remarkably, it is salient that seven studies associated with him have ascended to constitute the upper echelon of the ten most frequently cited articles within this field (15–21).

In light of these dynamics, collaborative networks of research materialize as instrumental conduits for researchers to augment the breadth of their investigative pursuits or to conjoin forces with cohorts engaged in cognate inquiries. Accordingly, a judicious author threshold of 107 was established. Employing VOSviewer, we proceeded to visualize the extant panorama and gradation of author interplay within this domain, with the ensuing outcomes being expounded in **Figures 6C, D**.

### 3.5 Journals

Upon analyzing the literature’s cited and citing journals, it was possible to determine the influential journals in the field. **Table 3** illustrated the top ten cited and citing journals, with DIABETES TECHNOLOGY & THERAPEUTICS holding the highest rank as the first citing journal, followed by DIABETES CARE and DIABETES RESEARCH AND CLINICAL PRACTICE. Among the cited journals, DIABETES CARE held the top spot, followed by DIABETES TECHNOLOGY & THERAPEUTICS and J DIABETES SCI TECHNOL. In 2022, DIABETES CARE held the highest impact factor among the citing journals, with a score of 17.152, followed by DIABETES TECHNOLOGY & THERAPEUTICS with a score of 7.337.

**Table 3:** The Top 10 citing and cited journals of publications on the clinical practice of CGM in diabetes mellitus from 2012 to 2022.

Rank	Citing Journals	Counts	2022 Journal Impact Factor	Rank	Cited Journals	Counts	2022 Journal Impact Factor
1	DIABETES TECHNOLOGY \& THERAPEUTICS	453	7.337	1	DIABETES CARE	2865	17.152
2	DIABETES CARE	190	17.152	2	DIABETES TECHNOLOGY \& THERAPEUTICS	2208	7.337
3	DIABETES RESEARCH AND CLINICAL PRACTICE	142	8.180	3	J DIABETES SCI TECHNOL	1547	0
4	PEDIATRIC DIABETES	114	3.409	4	DIABETOLOGIA	1538	10.460
5	DIABETES OBESITY \& METABOLISM	108	6.408	5	DIABETIC MED	1533	4.213
6	DIABETIC MEDICINE	98	4.213	6	NEW ENGL J MED	1490	176.079
7	DIABETES THERAPY	88	3.595	7	DIABETES	1380	9.337
8	FRONTIERS IN ENDOCRINOLOGY	70	6.055	8	DIABETES RES CLIN PR	1262	8.180
9	JOURNAL OF CLINICAL ENDOCRINOLOGY \& METABOLISM	55	6.134	9	JAMA-J AM MED ASSOC	1198	157.335
10	JOURNAL OF DIABETES INVESTIGATION	52	3.681	10	LANCET	1135	202.731

**Figure 7:**
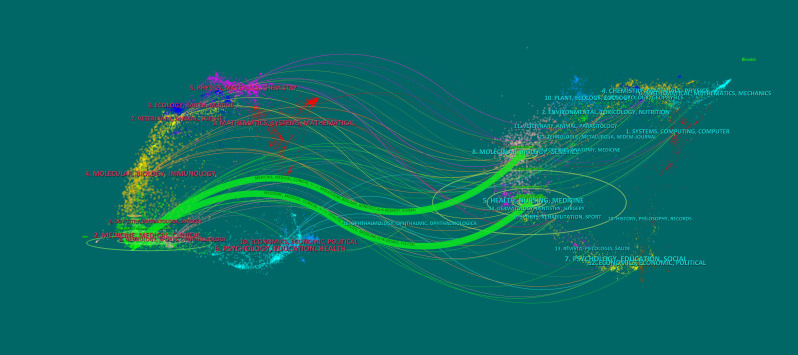
The Dual-map Overlay of Journals on the Clinical Practice of CGM in Diabetes Mellitus. The green path at the top suggests that research literature from MEDICINE, MEDICAL, CLINICAL area may be utilized to support the results and findings in the field of MOLECULES, BIOLOGY, GENETICS research. The findings from research literature in the MEDCINE, MEDICAL, CLINICAL area may be utilized to support the results from research conducted in the HEALTH, NURSING, MEDICINE area.

Furthermore, in plotting the Dual map overlay using CiteSpace, the journals that contributed to publications on the clinical practice of CGM in Diabetes Mellitus were analyzed. The resulting map in **Figure 7** was divided into two halves, with the left side representing the research area of the cited journals and the right side depicting the research area of the citing journals. The colored curves between the nodes on the left and right halves illustrated the relationship between the highly active research areas of the two journals. The examination of the graph indicated the presence of two discernible green curves, which implied that publications pertaining to medicine, medical and clinical domains have a higher likelihood of being referenced by journals that focus on molecular, biological, and genetic areas, as well as health, nursing, and medical fields.

### 3.6 Keywords

**Figure 8:**
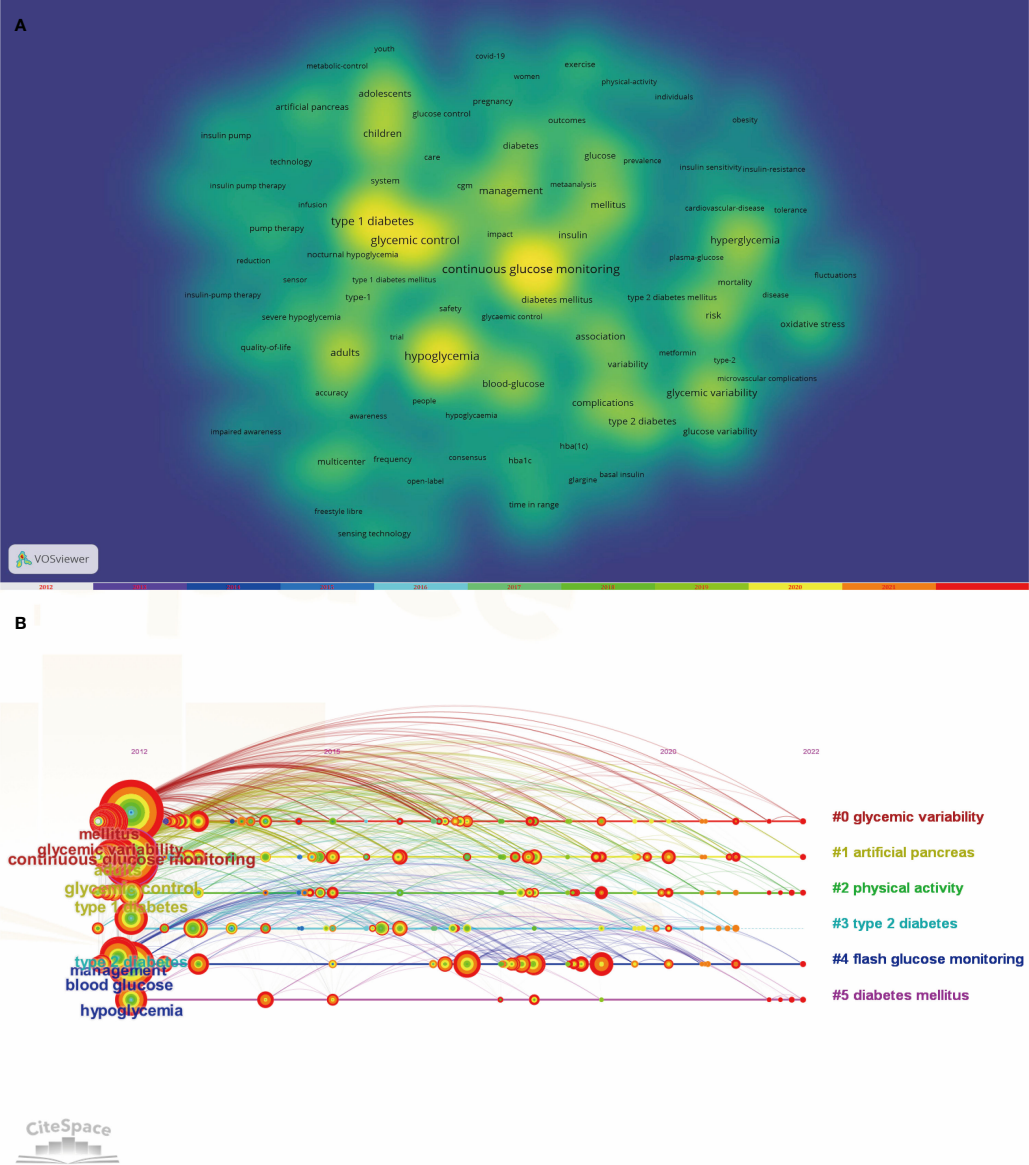
**(A)** Co-occurrence Keywords Network and Density Visualization on the Clinical Practice of CGM in Diabetes Mellitus from 2012-2022. **(B)** CiteSpace visualization timeline view of keywords clustering analysis related to the clinical practice of CGM in diabetes.

In this study, we utilized VOSviewer to visualize the 100 high-frequency keywords in the literature pertaining to the topic of interest. A threshold of 41 occurrences was set for the selection of these keywords. The resulting visualization in **Figure 8A** demonstrated that darker color blocks corresponded to higher frequency of occurrence of the respective keyword in the literature. Furthermore, proximity of a color block to the center of the yellow block indicated higher citation frequency and cited frequency. The top ten hot keywords, as ranked by frequency, were presented in **Table 4**. Notably, “Type 1 diabetes”, “Glycemic control”, “Hypoglycemia” were among the top keywords, suggesting their significance as hot topics in this research field over the past decade. And **Figure 8B** illustrates the chronological depiction of keyword clustering analysis, offering a timeline perspective. The diagram portrays various clusters denoted by distinctively colored horizontal lines on the right side, each corresponding to a collection of keywords. The nodes positioned along these horizontal lines symbolize individual keywords. Notably, the spatial arrangement of these nodes along the horizontal axis signifies the inaugural appearance year within the scholarly literature for the associated keyword, thereby constituting a comprehensive temporal representation of the keyword cluster’s evolutionary progression. The cluster “0# glycemic variability” is the largest, Next is “#1 artificial pancreas”, “#2 physical activity”, “#3 type 2 diabetes”, “#4 flash glucose monitoring” and “#diabetes mellitus”

**Table 4:** Top 10 keywords related to the clinical practice of CGM in diabetes mellitus from 2012-2022.

Rank	Keywords	Counts
1	Type 1 diabetes	683
2	Glycemic control	672
3	Hypoglycemia	523
4	Adults	410
5	Blood-glucose	352
6	Management	343
7	Glycemic variability	343
8	Children	307
9	Risk	301
10	Adolescents	299

**Figure 9:**
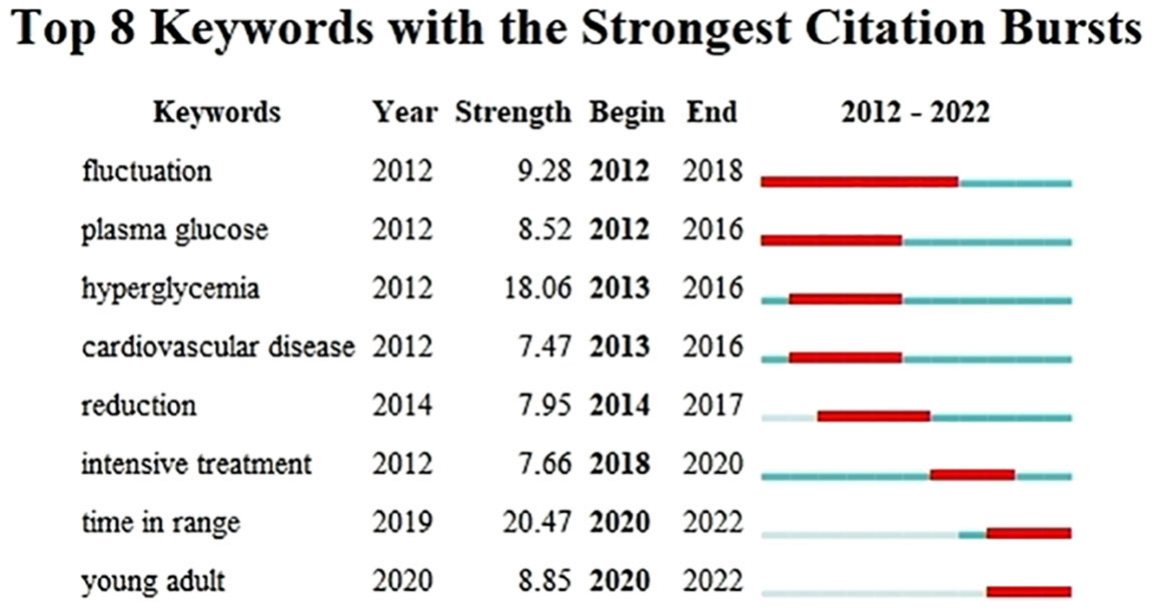
Keywords with the Strongest Citation Bursts for Publications on the Clinical Practice of CGM in Diabetes Mellitus Diabetic from 2012- 2022.

The CiteSpace is capable of identifying keywords that experience significant changes in frequency during a specific time period, commonly known as emergent words. Keywords that exhibit a delayed emergence and extended duration are indicative of the most recent research trends in a given field, enabling a temporal review of research hotspots and the projection of future trends. The default configuration of CiteSpace was substituted with the ensuing modes: “Year Per Slice” set to 1, “Top N%” set to 30.0%, and “Minimum Duration” set to 1. After conducting an analysis on the keywords with citation bursts, we determined that the 8 strongest burst keywords should be displayed as illustrated in **Figure 9**. During the period spanning from January 2012 to December 2022, the ensuing keywords surfaced as outcomes: fluctuation (2012-2018), plasma glucose (2012-2016), hyperglycemia (2013-2016), cardiovascular disease (2013-2016), reduction (2014-2017), intensive treatment (2018-2020), time in range (2020-2022), and young adults (2020-2022). In the preceding two years, the keywords “Time in Range” and “Young Adult” have surfaced and persisted throughout 2020 and beyond. Of the two, “Time in Range” has exhibited the most intense outbreak with a value of 20.47, indicating that it currently represents the primary research focus and potentially marks a pivotal juncture with notable implications for future inquiry.

### 3.7 Co-cited references

**Figure 10:**
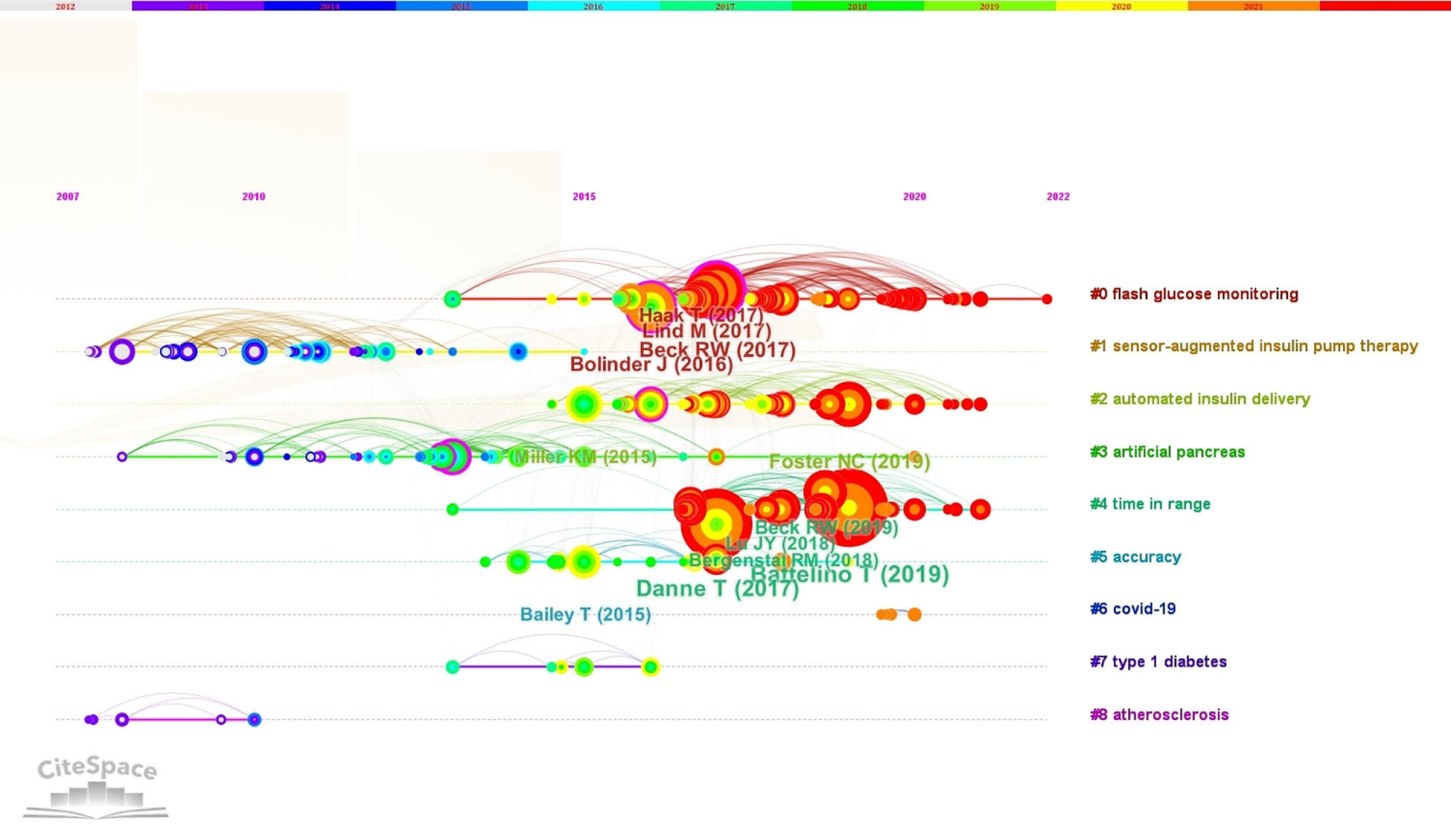
The Timeline View of Keyword Clustering Analysis Related to the Clinical Practice of CGM in Diabetes Mellitus was Visualized Using CiteSpace. The clusters formed by the keywords were represented by different colored horizontal lines, with labels on the right. The nodes positioned on these horizontal lines depicted the keywords themselves, while the position of the nodes on the horizontal lines indicated the year of the literature in which the keywords first appeared, thus forming a timeline representing the evolution of the keyword clusters.

A timeline map of co-cited references was constructed using CiteSpace with the aim of comprehending the principal research topics and their progression within the field. The outcomes of the keyword clustering analysis of the references were exhibited on the right-hand side of **Figure 10**, with “#flash glucose monitoring” comprising the most significant cluster. On the left-hand side, the citation relationship among each reference was presented over time, wherein larger nodes signified more frequent citations, and node color indicated the time when the reference was cited. The top 10 most frequently cited references were enumerated in **Table 5**.

**Table 5:** The Top 10 references of publications on the clinical practice of CGM in diabetes mellitus from 2012 to 2022.

Rank	Title of citing documents	DOI	Times cited	Interpretation of the research
1	Clinical Targets for Continuous Glucose Monitoring Data Interpretation: Recommendations From the International Consensus on Time in Range	doi: 10.2337/dci19-0028	557	This article summarizes the clinical practice of CGM in different populations, and if retrospective analysis of CGM data enables clinicians to set achievable clinical goals with their patients with diabetes and confirms that TIR is appropriate and useful in complementing clinical goals and outcome measures.
2	International Consensus on Use of Continuous Glucose Monitoring	doi:10.2337/dc17-1600	462	This article presents a synthesis of the consensus recommendations established by the Advanced Technologies and Treatments for Diabetes (ATTD) conference and serves as a comprehensive depiction of the contemporary comprehension regarding the potential impacts of continuous glucose monitoring (CGM) outcomes on clinical outcomes.
3	Effect of Continuous Glucose Monitoring on Glycemic Control in Adults With Type 1 Diabetes Using Insulin Injections The DIAMOND Randomized Clinical Trial	doi 10.1001/jama.2016.19975	291	A randomized controlled trial concludes that in patients with type 1 diabetes who receive multiple daily insulin injections, the use of CGM resulted in a significant decrease in HbA1c levels over 24 weeks compared to usual care.
4	Novel glucose-sensing technology and hypoglycaemia in type 1 diabetes: a multicentre, non-masked, randomised controlled trial	doi 10.1016/s0140-6736(16)31535-5	219	A multicenter, prospective, unmasked, randomized controlled trial concludes that the novel CGM reduces the duration of T1D hypoglycemia in adults.
5	Randomized Controlled Trial Continuous Glucose Monitoring vs Conventional Therapy for Glycemic Control in Adults With Type 1 Diabetes Treated With Multiple Daily Insulin Injections: The GOLD Randomized Clinical Trial	doi 10.1001/jama.2016.19976	200	This paper further validates the result that CGM reduces glycated hemoglobin
6	State of Type 1 Diabetes Management and Outcomes from the T1D Exchange in 2016–2018	doi 10.1089/dia.2018.0384	199	This article shows that only a small number of adults and youth with TID in the United States meet ADA goals
7	Validation of Time in Range as an Outcome Measure for Diabetes Clinical Trials	doi 10.2337/dc18-1444	177	This study provides validation that Time in Range (TIR) is highly correlated with the likelihood of microvascular complications and thus represents a valid endpoint for clinical trials.
8	Glucose Management Indicator (GMI): A New Term for Estimating A1C From Continuous Glucose Monitoring	doi 10.2337/dc18-1581	133	Introducing eA1C from CGM, a new glucose assessment metric for diabetes education or management
9	Continuous Glucose Monitoring Versus Usual Care in Patients With Type 2 Diabetes Receiving Multiple Daily Insulin Injections A Randomized Trial	doi 10.7326/m16-2855	132	Through a randomized controlled trial, the results of this study raise the possibility that CGM is potentially beneficial for adult patients with T2D who are treated with insulin, although CGM is rarely used.
10	Current State of Type 1 Diabetes Treatment in the U.S.: Updated Data From the T1D Exchange Clinic Registry	doi 10.2337/dc15-0078	131	An analysis of the data collected from 2010-2012 and 2013-2014 on T1D patients in the United States is conducted to provide insight into the current status of T1D patients.

## 4 Discussion

### 4.1 General information

In brief, the annual production of scholarly articles in this field exhibited an upward trend overall, with a significant and substantial surge observed in 2020, likely due to the outbreak of the COVID-19 pandemic. The pandemic-induced public health measures have altered people’s lifestyles, potentially impacting the glycemic control of individuals with diabetes by limiting physical activity to some extent (22). Furthermore, the use of glucocorticoid therapy may exacerbate hyperglycemia once severe infections such as COVID-19 pneumonia have developed. Consequently, a study has suggested that a system that integrates telemedicine and continuous glucose monitoring (CGM) can effectively manage blood glucose levels and prevent adverse outcomes (23, 24). As a result, CGM has gained increasing adoption in clinical settings, with a peak in related research output in 2021. In the analysis of countries within a particular research area, the United States emerged as the leading contributor in terms of number of publications, centrality, H-index, and citations. This suggested that the United States possessed a greater level of influence within this research area and engages in frequent collaborations with other countries. Based on the aforementioned analysis, it is recommended that research teams hailing from Asian countries seek to augment their international influence by engaging in heightened cooperation with their counterparts in European and American nations. The dual map overlay depicted in **Figure 7** reveals a wide range of subject areas covered by cited and citing journals, indicating untapped potential for further exploration within this research area. By employing a clustering analysis approach and examining the emergence of keywords and references, we have been able to identify research hotspots between 2012 and 2022 and forecast future trends in this research area. Notably, the most cited reference is a review authored by Tadej Battelino, Thomas Danne et al. This international consensus validated the feasibility of using the TIR index as a clinical endpoint and outcome measure to supplement HbA1c in various relevant populations, and the target thresholds outlined in the article serve as a valuable framework and point of reference for the clinical application of CGM (15).

### 4.2 Research hotspots

The fundamental essence of an academic field can be encapsulated by its keywords, and through visual analysis of these keywords, one can discern the prevailing research trends and trajectories (25). Based on the high-frequency keywords extracted and the keyword clustering timeline mapping generated by CiteSpace, the primary research areas in this field during the past decade can be identified. These areas include continuous glucose monitoring (CGM), glycemic variability, type 1 diabetes, and hypoglycemia. Significantly, the other three hot keywords were all generated based on CGM.

In the realm of diabetes management, the advent of continuous glucose monitoring (CGM) technology has bestowed unprecedented prospects for the monitoring and regulation of patient glycemic levels. Traditional intermittent approaches to glucose monitoring have progressively exhibited their inherent limitations, rendering the comprehensive capture of blood glucose fluctuations throughout a patient’s diurnal existence a challenging endeavor. In contrast, the real-time and uninterrupted monitoring attribute intrinsic to CGM technology introduces a novel instrument for therapeutic guidance, both for medical practitioners and their patients. The study of glycemic variability has emerged as a pivotal domain of investigation in contemporary times, and concomitant with the evolution of CGM technology, its integration within clinical practice has experienced a notable escalation in recent years (26, 27). Recent findings have indicated a correlation between elevated glycemic variability (GV) and the advancement and escalation of vascular complications in diabetic patients, heightened susceptibility to hypoglycemic episodes, as well as a decline in the quality of life (QOL) for affected individuals (28–30). The metrics encompassing Glycemic Variability are presently acknowledged as a significant gauge of glycemic management (31). This underscores the imperative of delving into the systematic examination of continuous glucose monitoring (CGM) data, and underscores the criticality of adeptly harnessing and interpreting CGM data to effectively serve its role within the realm of clinical practice.

The assessment of transient glycemic fluctuations is frequently derived from continuous glucose monitoring standard deviation (CGM.SD), a readily computable metric commonly employed to quantify short-term glycemic variability. However, it is worth noting that CGM.SD is influenced by the prevailing mean glucose levels, thereby rendering it susceptible to this parameter. Conversely, the coefficient of variation (CV), derived from both the standard deviation and mean glucose, serves to ameliorate this inherent limitation by compensating for the aforementioned sensitivity to mean glucose levels (15). Furthermore, within clinical settings, diverse indices such as the mean amplitude of glycemic excursion (MAGE), J-index, low blood glucose index/high blood glucose index, average daily risk range, and mean of daily differences (MODD) are employed to evaluate distinct facets of glycemic fluctuations in patients (32–36). Additionally, the Time in Range (TIR) parameter, denoting the proportion of time during which blood glucose levels remain within specified glycemic thresholds, while not strictly categorized as a glycemic variability metric, assumes significance as a supplementary clinical target and an outcome measure for HbA1c assessment across a spectrum of diabetes mellitus presentations, as established by international consensus (15). Consensus opinions have also established a link between TIR and the risk of diabetic complications, such as the close association between TIR and the risk of microvascular complications (19), as well as the good correlation between TIR and HbA1c. Additional research has further substantiated the notion that HbA1c inadequately captures data pertaining to the fluctuation of blood glucose levels or the duration of time spent within the hypoglycemic or hyperglycemic spectrum. Consequently, the Time in Range (TIR) metric is presently being embraced as a favored metric for prognosticating the susceptibility to diabetic complications, delineating outcomes in clinical investigations, and evaluating glycemic management in patient cohorts (37, 38).

As the keyword emergence shown in **Figure 9**, the emerging keywords for 2020 to 2022 are “Time in Range” and “Young Adult”. Research conducted in the realm of clinical practice concerning Continuous Glucose Monitoring (CGM) throughout this timeframe has been predominantly centered around acquiring more precise and up-to-the-minute data pertaining to glycemic regulation. Additionally, investigations have been dedicated to the viability of employing Time in Range (TIR) as a quantifiable parameter, alongside examinations concentrated on the demographic of young adults. Envisioning the future, the burgeoning prominence of TIR as a measurable criterion is anticipated to persistently mirror the evolving methodology within the domain of diabetes management. Progressions in technological innovations coupled with an increasingly profound comprehension of the intricacies inherent in diabetes are poised to propel these transformations forward, culminating in a sustained emphasis on ameliorating long-term prognosis and enhancing the quality of life for individuals afflicted with diabetes.

Although Continuous Glucose Monitoring (CGM) can furnish real-time insights into blood glucose levels and trends, alongside retrospective analyses of glycemic regulation patterns and glycemic metrics over specific temporal intervals (4, 39), their assimilation into clinical practice falls short of reaching optimal levels (40). The suboptimal adoption can be attributed, in significant part, to the dearth of software possessing the capacity for relatively straightforward and standardized statistical and graphical depiction, as well as interpretation, of glycemic data, thereby engendering uncertainty and reluctance among clinicians towards integrating CGM into their professional milieu (41, 42). Consequently, to surmount these obstacles and harness the full potential of continuous glucose monitoring (CGM) data within clinical settings, a method christened as the “ambulatory glucose profile” (AGP) was devised. The AGP is a tool utilized for assessing short-term glycemic variability indices in diabetic patients. By analyzing CGM data, it generates charts depicting median, interquartile range (IQR), and other statistical values, thereby providing a comprehensive evaluation of intra-day and inter-day glycemic fluctuations for patients (43, 44). A methodical examination of AGP reports proves to be a valuable and pragmatic approach, enabling real-time and comprehensive assessment of glycemic control and the effectiveness of any therapeutic modifications (45, 46). Through meticulous scrutiny of AGP charts, clinical practitioners can attain enhanced comprehension of patients’ glycemic patterns, identify potential issues, and discern opportunities for refining treatment regimens. Thorough AGP analysis aids in pinpointing pivotal factors for achieving optimal glycemic control, thereby furnishing robust support for formulating appropriate therapeutic adjustments and further integrating CGM data into routine clinical practice (47, 48). With the introduction of the AGP approach, clinicians are better equipped to expound upon and communicate glycemic data, collaboratively establish personalized treatment objectives with patients, and monitor their progress throughout the course of treatment. This endeavor fosters closer doctor-patient relationships, heightens patients’ awareness of glycemic control, and ultimately augments the efficacy of diabetes management. In addition, there are a number of Software Packages and Tools that support comprehensive analysis of CGM data (49).

Ultimately, through a bibliometric analysis of this research domain, we can discern with clarity that the focal point of clinical practice research transcends the mere analysis of various metrics and has surpassed conventional data analysis. As depicted by the keyword clustering analysis in **Figure 8**, investigations are progressively expanding into the application of cutting-edge technologies such as artificial pancreas, machine learning, and artificial intelligence. These studies not only furnish diabetic patients with more advanced therapeutic modalities but also usher in novel possibilities for technological innovation and advancement within the realm of medicine. Looking ahead, we can anticipate witnessing further breakthroughs in these domains, heralding a positive impact on the well-being and quality of life for individuals afflicted by diabetes.

## 5 Conclusion

The current clinical practice of continuous glucose monitoring (CGM) holds great promise. Since its introduction in the United States in 1999 (50), the accuracy of CGM systems has steadily improved, facilitating better daily management of diabetes. The present state of glycemic management in diabetic patients is deemed precarious, as it falls short of the established standards set forth by the World Health Organization. Nevertheless, the burgeoning utilization of continuous glucose monitoring (CGM) has garnered significant interest and is anticipated to be comprehensively explored in the realm of research.

## 6 Limitations

The present study exhibits certain potential limitations that should be acknowledged. Firstly, the pertinent articles were exclusively obtained from a solitary database, WOSCC, which may have resulted in a biased sample, particularly in comparison to other databases, such as Scopus and PubMed. Secondly, some studies that could have provided valuable insights to the study are ongoing and hence not yet included. Thirdly, researcher bias is a possibility, as the screening process for literature necessitates the artificial exclusion of articles that do not bear relevance to the study. Fourthly, the study solely focused on the clinical practice of CGM in diabetes and did not encompass the technological advancements of CGM sensors, which may have caused the omission of certain potentially beneficial articles.

## Data availability statement

The raw data supporting the conclusions of this article will be made available by the authors, without undue reservation.

## Author contributions

LK, BD, and MG contributed to conception and design of the study. LY and ZX organized the database. BD performed the statistical analysis. LK and BD wrote the first draft of the manuscript. MG, MC, XW, MZ, HT, and QW wrote sections of the manuscript. All authors contributed to the article and approved the submitted version.
